# Peroxisome proliferator-activated receptor (PPAR) α and δ activators induce ICAM-1 expression in quiescent non stimulated endothelial cells

**DOI:** 10.1186/s12950-016-0135-2

**Published:** 2016-08-20

**Authors:** Julia Naidenow, Igor Hrgovic, Monika Doll, Tsige Hailemariam-Jahn, Victoria Lang, Johannes Kleemann, Stefan Kippenberger, Roland Kaufmann, Nadja Zöller, Markus Meissner

**Affiliations:** Department of Dermatology, Venereology and Allergology, Johann Wolfgang Goethe-University, Theodor-Stern-Kai 7, D-60590 Frankfurt am Main, Germany

**Keywords:** PPARα, PPARδ, mRNA stability, ICAM-1, Endothelial cell, Sp1, Promoter

## Abstract

**Background:**

Peroxisome proliferator-activated receptors (PPARs) are ligand-activated transcription factors that are implicated in the regulation of lipid and glucose homeostasis. PPAR agonists have been shown to control inflammatory processes, in part by inhibiting the expression of distinct proinflammatory genes such as vascular cell adhesion molecule-1 (VCAM-1), IL-8, and intercellular adhesion molecule-1 (ICAM-1). ICAM-1 is an important endothelial membrane receptor that facilitates the transmigration of leukocytes across the endothelium. To date, the influence of PPARα and δ activators on the expression of ICAM-1 in non-induced, quiescent endothelial cells has been unclear. Therefore, we examined the effects of various PPARα and δ agonists on the expression of ICAM-1 in non-stimulated primary human endothelial cells.

**Results:**

We found that PPARα and PPARδ agonists significantly induced ICAM-1 surface, intracellular protein, and mRNA expression in a time and concentration-dependent manner. The PPARδ induced ICAM-1 expression could be paralleled with a significantly increased T-cell adherence to the endothelial cells whereas PPARα failed to do so. Transcriptional activity studies using an *ICAM-1* reporter gene constructs revealed that PPARδ, but not PPARα agonists induced gene expression by stimulating *ICAM-1* promoter activity via an Sp1 transcription factor binding site and inhibit the binding of the transcription factors Sp1 and Sp3. Furthermore, we performed mRNA stability assays and found that PPARα and PPARδ agonists increased *ICAM-1* mRNA stability.

**Conclusion:**

Therefore, our data provide the first evidence that PPARα and PPARδ agonists induce ICAM-1 expression in non-stimulated endothelial cells via transcriptional and posttranscriptional mechanisms.

## Background

Peroxisome proliferator-activated receptors (PPARs) are members of the nuclear receptor-activated transcription factor superfamily comprised of three subtypes: PPARα, PPARδ, and PPARγ. The role of PPARs was originally thought to be restricted to lipid metabolism, glucose homeostasis, and cellular differentiation [1–4]. PPARs can be activated by natural ligands such as eicosanoids or fatty acids. In addition, synthetic antidiabetic thiazolidinediones and lipid-lowering fibrates have been shown to act as activators of PPARγ and PPARα, respectively [5–7]. Recent evidence suggests that PPARδ plays a crucial role in the regulation of differentiation, cell growth, and the metabolism of lipids and glucose [8–11]. Previous studies demonstrated that PPARδ agonists improve insulin sensitivity and therefore might be interesting targets for the treatment of obesity-associated disorders [12–14].

In the last few years, several studies have revealed the impact of PPARα and PPARδ on endothelial cell function. During inflammation, proinflammatory stimuli, including LPS, TNFα or IL-1β, cause phenotypic changes to the quiescent endothelium by inducing the expression of proinflammatory factors such as IL-6 and IL-8 or adhesion molecules such as ICAM-1 and VCAM-1. Fan et al. demonstrated that the PPARδ agonist GW501516 suppressed IL-1β-induced VCAM-1 and E-selectin expression in human umbilical vein endothelial cells (HUVECs) [15]. Furthermore, Rival et al*.* showed that the PPARδ activator L-165041 suppressed TNFα-induced VCAM-1 and MCP-1 expression [16]. Huang et al*.* revealed that ICAM-1 expression could be attenuated by PPARα activation via fenofibrate in TNFα - activated human aortic endothelial cells [17]. Piqueras demonstrated, that TNFα induced ICAM-1 expression in endothelial cells can be partly suppressed by PPARδ agonists [18]. Taken together, these studies demonstrate that PPARα/δ agonists play a role in suppressing the proinflammatory response in stimulated endothelial cells.

On the other hand, there is also evidence that PPAR agonists have proinflammatory properties in non-inflammatory, quiescent endothelial cells. Chen et al*.* demonstrated that PPARγ agonists significantly induced ICAM-1 expression in human endothelial cells [19]. In addition, PPARδ agonists were shown to induce IL-6 and IL-8 expression in non-stimulated human endothelial cells via increased induction and translocation of NfkB [20]. Recently, Gu et al*.* demonstrated, that the PPARα agonists fenofibrate induces inflammation in experimental acute colitis mice [21]. Furthermore, Wang et al*.* demonstrated that PPARδ promotes colonic inflammation and colitis-associated tumor growth via the COX-2-derived PGE2 signaling [22]. Therefore, it is important, that not only the anti-inflammatory action of PPARs but also the possible pro-inflammatory properties are investigated.

The impact of PPARα and PPARδ agonists on ICAM-1 expression in quiescent non stimulated endothelial cells has yet to be assessed. Understanding the consequences of PPAR signaling is of importance due to the possible wide range use of PPAR agonists in various diseases such as chronic inflammation, glucose metabolism, dyslipidemia, obesity, cancer therapy, and potentially many more. In the present study, we analyzed the effects of PPARα and PPARδ activators on the expression of ICAM-1 in non-stimulated HUVECs. Furthermore, we investigated the mechanisms by which PPAR agonists exert their influence within these cells.

## Methods

### Reagents

Recombinant human TNFα was purchased from R&D Systems (Minneapolis, MN, USA). L-165041, GW501546, WY14643, Fenofibrate and Actinomycin-D were obtained from Sigma-Aldrich (Hamburg, Germany).

### Cell culture

HUVECs were purchased from PromoCell (Heidelberg, Germany) and were cultured until the fifth passage at 37 °C and 5 % CO_2_ in Endothelial Cell Growth Medium (Cambrex, East Rutherford, NJ, USA). Jurkat cells were obtained from ATCC (LGC Standards; Wesel, Germany) and cultured at 37 °C and 5 % CO_2_ atmosphere in RPMI-1640 medium supplemented with 10 % fetal bovine serum (FBS) and 2 mM glutamine, 100 U/ml penicillin, and 100 μg/ml streptomycin.

### Fluorescence-activated cell sorting analysis

HUVECs were treated with PPARα and PPARδ agonists for 24 h. The treated cells were incubated with mouse anti-human ICAM-1 fluorescein-conjugated mAb (Clone #BBIG-1; 1:200 dilution; R&D Systems, Wiesbaden, Germany) or isotype control mouse anti-human IgG1 (R&D Systems, Wiesbaden, Germany) for 30 min on ice. Isotype control cells were then incubated with fluorescein-conjugated affinity-purified goat F(ab´)2 anti-mouse IgG (F0479; DAKO, Hamburg, Germany) at a 1:100 dilution for 30 min, and cells were subsequently analyzed by a BD FACScan Cytometer (Becton–Dickinson, Franklin Lakes, NJ, USA). Nonviable cells were identified and excluded by propidium iodide staining.

### Western blot analysis

Whole cell protein was prepared as previously described [23]. Membranes were incubated with the indicated primary antibodies. Antibodies were as follows: anti-ICAM-1 (SC-107; 15.2) from Santa Cruz (Heidelberg, Germany) and anti-Tubulinα Ab-2 (DM1A) from LabVision (Fremont, CA, USA). Primary antibody application was followed by incubation with horseradish peroxidase-conjugated secondary antibodies (anti-mouse and anti-rabbit IgG, Amersham, Uppsala, Sweden; anti-goat, Dako, Glostrup, Denmark). Blots were developed using an enhanced chemiluminescence detection system (ECL) (Amersham, Uppsala, Sweden), according to the manufacturer’s instructions. Densitometry was used to quantify band intensities using ImageJ (v1.29 s). Optical densities of the bands were corrected for loading differences based on corresponding control bands.

### Flow chamber assays

Adhesion was determined using Ibidi μ-slide VI chambers (Munich, Germany) [24]. Two days before treatment 1,8x10^5^ HUVECs were seeded in each chamber. The day before treatment HUVECs were serum-starved (0,5 % FBS) for 24 h. After 24 h the HUVECs were treated with PPARδ or PPARα agonists (L-165,041 (50 μM); GW501516 (20 μM); Fenofibrate (100 μM); WY14643 (200 μM)) or TNFα (20 ng/ml) for 6 h. 5x10^4^ Jurkat cells were allowed to attach on the endothelium for 3 min. Non-adherent cells were flushed away and shear stress was increased stepwise from 0.35 to 2, 5, 8 and 15 dyn/cm2 for 30 s each. Every 30 s the number of adherent cells was quantified, with a charge-coupled device (CCD) camera (Sony, New Jersey, USA).

### RNA extraction and RT-PCR

RT-PCR analyses were performed using total RNA (150 ng) extracted from sub-confluent cell cultures. Total cellular mRNA was isolated by the RNeasy Mini Procedure (Qiagen, Hilden, Germany) after DNase digestion. RT-PCR analyses for ICAM-1 and GAPDH were performed using the One Step RT-PCR Kit (Qiagen). PCR products were resolved by 1–2 % agarose gel electrophoresis, and ethidium bromide-stained bands were visualized with an ultraviolet transilluminator. The primer sets for ICAM-1 and GAPDH have been previously published [23]. Densitometry was used to quantify band intensities using ImageJ (v1.29 s). Optical densities of the bands were corrected for loading differences based on corresponding control bands.

### Transient transfection and analysis of reporter gene expression

HUVECs (1.0 × 10^5^ cells/well in 12-well plates) were transfected with 0.5 μg of the appropriate *firefly* luciferase construct and 0.1 μg phRG-TK vector (Promega, Madison, WI, USA) using the SuperFect transfection reagent (Qiagen). Human ICAM-1 full length reporter gene construct -1014 pIC was generously provided by Paul van der Saag, Hubrecht Laboratorium, Utrecht, The Netherlands and sublconed in a pGL3 luciferase vector (Promega, Madison, WI, USA) [25]. In addition, four new deletional ICAM-1 promoter constructs containing 5′regulatory elements were established in the pGL3 luciferase vector using PCR amplification (HotStar HiFidility Polymerase Kit; Qiagen) and KpnI and BglII restriction sites. The −1014 gene construct was used as a template. All constructs were sequenced from the 5′- and 3′-ends to confirm orientation and sequence correctness. Twenty-four hours after transfection, cells were treated with vehicle (DMSO, 0.3 %) or the appropiate PPAR agonist for 24 h. Luciferase activities were measured with the Dual-Luciferase Reporter Assay System (Promega).

### Preparation of nuclear extracts and electrophoretic mobility shift assay (EMSA)

HUVECs were treated with vehicle (0.1 % DMSO) or L-165041 for 30 min. Nuclear proteins were extracted as described previously [26]. An ICAM-1 promoter specific oligonucleotide between −69 and −45 bp containing the Sp1 site between −53 and −59 bp was constructed (5′primer: GAAAGCAGCA**CCGCCC**CTTGGCCC (Sp1-site in bold letters); 3′primer: GGGCCAAGG). In addition, a Sp1 mutated olignucleotoide was constructed carrying two nucleotide mutations within the Sp1 consensus sequence (5′primer: GAAAGCAGCA**C*A*G*A*CC**CTTGGCCC (mutated Sp1-site in bold letters, exchanged nucleotides in italic); 3′primer: GGGCCAAGG). DNA-binding reactions were performed with or without excess unlabeled competitor, Sp1 consensus-oligonucleotide (Promega), wildtype Sp1 oligo and mutated Sp1 oligo as well as Sp1 and Sp3 antibodies (SC-59 (PEP2); SC644 (D-20)Santa Cruz Biotechnology, Santa Cruz, CA).

### Statistical analyses

The data are expressed as means ± SEM from at least three independent experiments. Statistical analyses were performed using the ANOVA test.

## Results

### PPARα and δ agonists induce ICAM-1 surface and whole cell protein expression in non-stimulated human endothelial cells

**Fig. 1:**
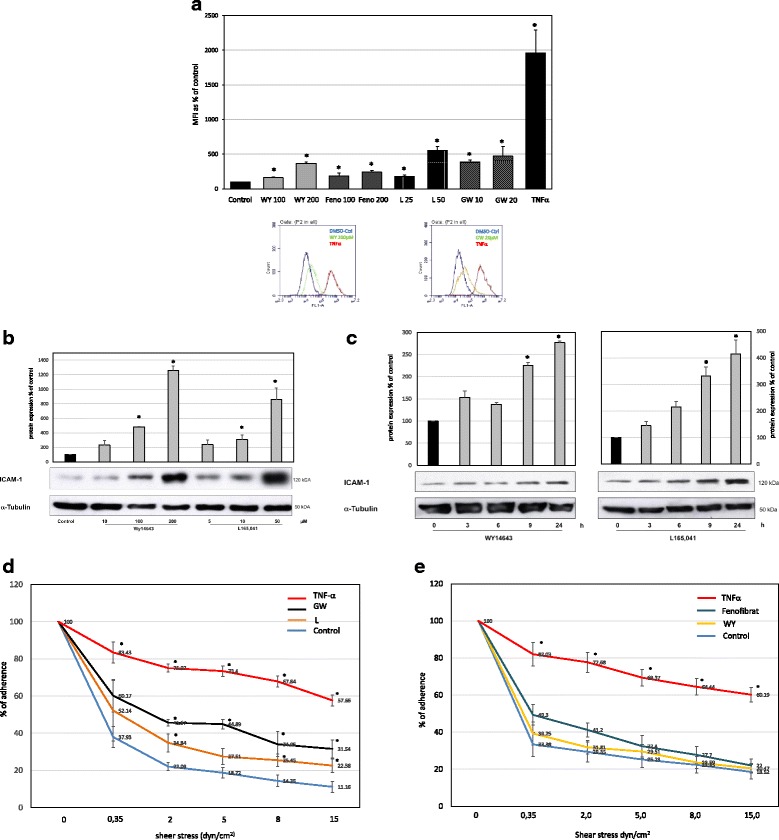
Effects of PPARα and δ agonists on the ICAM-1 surface and protein expression as well as funtion in non-stimulated HUVEC. **a** Flow-cytometric analyses of ICAM-1 expression; HUVECs were left untreated (solvent only) or were treated with different concentrations of PPARα agonsist (WY14643 (100 and 200 μM) and Fenofibrate (100 and 200 μM) or PPARδ agonists (L-165041 (25 and 50 μM) and GW501514 (10 and 20 μM)). As positive control TNFα (20 ng/ml) was used. Mean values from triplicate experiments performed four times are depicted ± SEM. *p < 0.05 was considered significant. Exemplary plots for the PPARα agonist WY14643 and the PPARδ agonist GW601514 are depicted. **b** Representative western blot analyses of endothelial cells that were left untreated (solvent only) or were treated with WY14643 and L-165041 for 24 h at different concentrations. Comparable results were obtained from three independent experiments. The results were normalized to the expression of tubulin. The relative expression of ICAM-1 is presented in % of control. The mean values from three independent experiments are presented as the mean ± SEM. *p < 0.05. **c** Representative western blot analyses of endothelial cells that were left untreated (solvent only) or were treated with WY14643 (200 μM) and L-165041 (50 μM) at different time points. Comparable results were obtained from three independent experiments. The results were normalized to the expression of tubulin. The relative expression of ICAM-1 is presented in % of control. The mean values from three independent experiments are presented as the mean ± SEM. *p < 0.05. **d** Adhesion assay for the functional interaction between T cells (Jurkat) and endothelial cells: HUVECs were left untreated (solvent only) or were treated with PPARδ agonists for 24 h (L-165041 (50 μM) and GW501514 (20 μM)) or (**e**) PPARα agonists for 24 h (Fenofibrat (100 μM) and WY14643 (200 μM)). As positive control TNFα (20 ng/ml) was used. Jurkat cells were allowed to adhere to the endothelial cells for 3 min following stepwise increase of shear stress. The number of adherent cells was quantified. The mean values from five independent experiments are presented as the mean ± SEM. *p < 0.05 was considered significant

We performed FACS analysis to evaluate the effects of the PPARα agonists fenofibrate and WY14643 as well as the PPARδ agonists L165041 and GW501516 on the surface expression of ICAM-1 in non-stimulated, quiescent HUVECS. The concentrations of the PPAR agonists were used as published and do not induce any cytotoxicity as previously shown [12, 20, 26–29]. Treatment of non-stimulated HUVECs with each agonist resulted in an induction of ICAM-1 surface expression (Fig. 1a). The level of induction was comparable between the two agonists of each PPAR, indicating that the observed effects are specific to PPARα and PPARδ. We also examined the effects of PPARα and PPARδ agonists on whole cell protein expression of ICAM-1. Western blot analysis of whole-cell extracts demonstrated that both PPAR agonists induced ICAM-1 expression in a concentration and time-dependent manner (Fig. 1b,c). To analyze whether the general ICAM-1 induction on endothelial cells has a functional consequence, we performed adhesion assays with the T-cell line Jurkat and PPARδ and PPARα agonists treated HUVEC as a proof of concept (Fig. 1d,e). Here we could demonstrate, that the PPARδ agonist induced ICAM-1 expression elevates the adherence of Jurkat cells significantly whereas PPARα agonists failed to increase T-cell adherence.

### PPARα and δ agonists induce *ICAM-1* steady-state mRNA expression

**Fig. 2:**
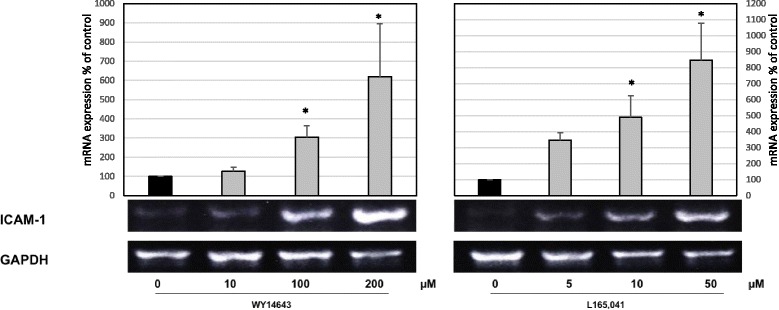
Effects of PPARα and δ agonists on the ICAM-1 mRNA expression. RT-PCR analyses of total mRNA extracted from HUVECs that were treated with vehicle (solvent only) or L165041 and WY14643 for varying concentration as indicated for 24 h. Results were confirmed in three independent sets of experiments. The results were normalized to the expression of GAPDH. The relative expression of ICAM-1 is presented in % of control. The mean values from four independent experiments are presented as the mean ± SEM. *p < 0.05

We examined whether PPARα and PPARδ agonists affect the steady-state mRNA levels of ICAM-1 in HUVECs. Consistent with our protein expression data, treatment with both PPARα and PPARδ agonists induced *ICAM-1* mRNA expression in a concentration-dependent manner (Fig. 2).

### PPARδ, but not PPARα agonists upregulate *ICAM-1* promoter activity via a Sp1 binding site between −59 and −53 bp

**Fig. 3:**
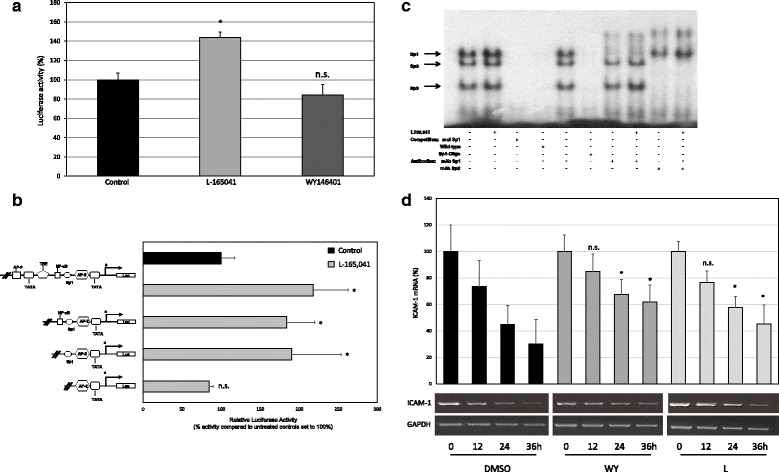
Effects of PPARα and δ agonists on the ICAM-1 promoter activity and mRNA half-life. **a** Analyses of wildtype ICAM-1 luciferase (Luc) reporter construct in HUVECs. The Luc activity is expressed in percent of control (mean ± SEM of three independent experiments each performed in triplicate). HUVECs were left untreated (solvent only) or were treated with different concentrations of PPARα agonsist (WY14643 (200 μM) and PPARδ agonists (L-165041 (50 μM)) for 24 h. As positive control TNFα (20 ng/ml) was used. Mean values from triplicate experiments with four independent experiments are depicted ± SEM. *p < 0.05 was considered significant. **b** Analyses of 5′-deletional ICAM-1 promoter-based luciferase constructs in HUVECs. Schematic representation of the respective reporter gene constructs on the left and the relative Luc activities (expressed as % activity of the control cells) in graphic format on the right. HUVECs were left untreated (solvent only) or were treated with the PPARδ agonists (L-165041 (50 μM)) for 24 h. Results were confirmed in four independent sets of experiments. *p < 0.05. **c** Representative EMSAs using nuclear extracts of HUVECs that were left untreated (solvent only) or were treated with PPARδ agonists L-165041 (50 μM) for 24 h (lane 1,2): mutated labelled Sp1 DNA (lane 3), competition with unlabelled wild-type DNA (lane 4, at 100 molar excess) or with unlabelled excess double-stranded Sp1 consensus oligonucleotides (lane 6, at a final concentration of 0.35 lmol ⁄ l). Supershift analyses were performed by addition of specific Sp1 (lane 7 and 8) or Sp3 antibody (lanes 9 and 10, all from Santa Cruz)) at a final concentration of 100 ng ⁄ ml. Formation of Sp-dependent binding complexes is indicated by arrows to the left. A representative autoradiography from three independent experiments is shown. **d** HUVEC were incubated with vehicle, L165041 (50 μM) or WY14643 (200 μM) for 1 h, followed by incubation with fresh media containing Act D (10 μg/ml) for 0, 12, 24 and 36 h. RT-PCR analyses for ICAM-1/GAPDH of total RNA extracted from subconfluent cell cultures were performed. The PCR products were separated by 2 % agarose gel electrophoresis, and ethidium bromide stained bands visualized using an ultraviolet transilluminator. ICAM-1 bands were quantified by densitometric scanning, the results of which were normalized to amounts of GAPDH mRNA. The mean values from five independent experiments are presented as the mean ± SEM. *p < 0.05

We hypothesized that PPARα and PPARδ agonists induce *ICAM-1* expression through transcriptional regulation of the *ICAM-1* promoter. To test this possibility, a luciferase reporter construct containing all transcription factor binding sites of the *ICAM-1* promoter was transiently transfected into vehicle- and agonist-treated HUVECs. Analysis of luciferase expression revealed a significant 1,4-fold induction of *ICAM-1* promoter activity in response to PPARδ agonist. However, PPARα agonist treatment failed to activate transcriptional activity of the *ICAM-1* promoter (Fig. 3a). To further analyze the underlying mechanisms of PPARδ agonist induced *ICAM-1* promoter activity a series of *ICAM-1* promoter deletions was introduced into a luciferase reporter construct and transiently transfected into HUVECs (Fig. 3b). Interestingly, the deletion of a Sp1-binding site located between −59/ −53 bp led to a complete loss of PPARδ agonist induced *ICAM-1* promoter activity. In contrast, constructs containing this site demonstrated a significant induction of *ICAM-1* promoter acitivity during PPARδ agonist treatment. Therefore, this single Sp1 site seems to be responsible for PPARδ agonist conveyed *ICAM-1* promoter activity.

### PPARδ agonists induce SP1 and Sp3 transcription factor binding to the Sp1 binding site at −59/−53 bp

To determine the nuclear factor binding to the Sp1 binding site we performed EMSAs using nuclear extracts from control and PPARδ agonist treated HUVEC, a wildtype Sp1-oligonucleotide, a mutated Sp1-oligonucleotide and a consensus Sp1 oligonucleotide (Fig. 3c). Here we could demonstrate that the treatment with PPARδ agonists induces transcription factor (TF) binding to the Sp1 binding site. Via supershift analysis we could show, that not only Sp1 but also Sp3 TFs bind to the wildtype ICAM-1 Sp-1 binding site between −59/−53 bp. A mutation of the Sp1 binding site abolished the binding of the TFs.

### PPARα and PPARδ agonists induce *ICAM-1* mRNA stability

*ICAM-1* expression is not only controlled on the transcriptional level, but can also be influenced by mRNA stability. We therefore used actinomycin-D, a transcription inhibitor, to determine whether PPARα or δ agonists treatment increased *ICAM-1* mRNA stability in HUVECs. We found that *ICAM-1* mRNA stability was significantly increased in PPARα as well as PPARδ agonist treated cells (Fig. 3d).

## Discussion

Insights into the function of PPAR activators have rapidly grown over the past decade. Recently, the impact of PPAR agonists especially in endothelial cell function and angiogenesis has been addressed [27–31]. These findings are critical for uncovering the breadth of PPAR functions in multiple cellular processes as PPAR agonists become increasingly more prevalent in clinical practice. Important pro-inflammatory cytokines, such as TNFα, IL-1β, and LPS, are known to induce ICAM-1 expression, and PPAR agonists are capable of potently suppressing these inflammatory effects [32–35]. Therefore, PPAR agonists are promising anti-inflammatory compounds for conditions such as chronic inflammatory diseases, cancer or obesity. However, the effects of PPAR activation in a non-inflammatory setting have not been previously addressed. This is an important consideration as PPAR agonists enter clinical testing for treatment and preventative strategies in various non-inflammatory diseases.

In the current study, we demonstrated that PPARα and PPARδ agonists effectively induced ICAM-1 expression in non-stimulated, quiescent endothelial cells. We identified two mechanisms underlying this effect: a transcriptional promoter-based mechanism for PPARδ agonists via increased binding of the TF Sp1/Sp3 at the ICAM-1 promoter between −59/53 bp and a posttranscriptional mechanism mediated by increased mRNA stability for PPARα and PPARδ agonists. In addition, we could demonstrate, that the increase in ICAM-1 surface expression leads to an increased T-cell adherence in PPARδ treated cells. Therefore, the current work supports the possibility of a dual function of the PPARα and δ agonists in endothelial cells that is dependent on the activation status of the cell. This novel information may have important implications for the responsiveness of different disease types to treatment with these compounds. The dual action of PPAR agonists may also account for the variable effects of PPAR activation in the treatment and development of distinct tumor entities [36–38]. It is possible that the activation status of the cancer cells or tumor stromal cells, including macrophages, fibroblasts, endothelial cells, and others, may influence PPAR function.

Concerning adipocytes and endothelial cells such a dual mechanism has been already described. Rodriguez-Calvo et al*.* demonstrated a reduced IL-6 expression after the previously LPS-stimulated adipocytes were treated with the PPARδ activator GW501516 [39]. The same group also showed an increase of IL-6 mRNA and protein in the control group treated only with the PPARδ agonist. Chen et al*.* demonstrated, that PPARγ agonists significantly induce ICAM-1 expression in non stimulated human vascular endothelial cells [19]. In addition, it could be demonstrated that PPARδ agonists induce IL-6 and IL-8 expression in non-stimulated human endothelial cells [20]. On the other hand Piqueras et al. demonstrated in TNFα stimulated endothelial cells a suppression of the induced ICAM-1 expression via PPARδ agonist treatment [18].

Our results further demonstrated that PPARα and PPARδ agonists influences *ICAM-1* mRNA stability. The regulation of *ICAM-1* mRNA stability is a well-accepted and important mechanism underlying posttranscriptional control of *ICAM-1* gene expression [40, 41]. PPARα agonists have already been implicated in regulating mRNA stability of other genes. Ren et al*.* demonstrated that activation of PPARα resulted in reduced *nephrin* mRNA stability, and therefore a decrease in *nephrin* expression in kidney epithelial cells [42]. Meissner et al*.* showed that *IL-8* mRNA stability was increased by PPARδ agonist treatment [20]. These results demonstrate the various, and likely context-dependent, mechanisms by which PPAR agonists regulate gene expression.

Besides mRNA stability, we could demonstrate that PPARδ agonists induce promoter activity via increased binding of the TFs Sp1/Sp3 at the Sp1 binding site in the proximal ICAM-1 promoter. Sp1 is one of the important transcription factors of ICAM-1 expression in endothelial cells. Brendji-Grün et al*.,* demonstrated in murine aortic endothelial cells that IL-1β induces ICAM-1 experession via critical Sp1 binding sites [43]. Kornschnabl et al*.* showed that the Sp1 site between −59 and −53 bp is essential for the ICAM-1 induction in cytomegalovirus infected HUVEC [44]. Furthermore, Zhang et al recently demonstrated that melanoma CD44 engagement wit endothelial E-selectin leads to the induction of ICAM-1 via increased binding of the TF Sp1 on the ICAM-promoter [45]. Therefore, Sp1 is an important TF for ICAM-1 regulation. We could demonstrate that the binding of Sp1/Sp3 on the Sp1 site is essential for PPARδ induced ICAM-1 transcriptional expression. Recently, Okazaki et al*.,* demonstrated that PPARδ agonists increase human SIRT-1 transcription via increased Sp1 promoter binding [46]. Comparable results were demonstrated from Bonofiglio et al*.,* who showed that rosiglitazone, a PPARγ agonist, induces fas ligand promoter activity via an increased Sp1 promoter binding [47]. Hong et al. demonstrated a comparable mechanism of PPARγ dependent p21 increase via Sp1 dependent p21 promoter activity [48].

Interestingly, only the induction of ICAM-1 expression by PPARδ agonists increased T-cell adhesion. PPARα agonists failed to increase T-cell adherence. This might be explained by the influence of PPARα agonists on the expression of further adhesion molecules overriding the effect of ICAM-1. Nevertheless, the induction of ICAM-1 by PPARα agonists might be relevant in a multi-drug setting. It could be demonstrated that besides the facilitating of leukocyte trans-endothelial migration ICAM-1 can for example bind fibrinogen preventing endothelial cell apoptosis and influencing vasomotorical reactions [49].

## Conclusion

Taken together, we have shown that treatment with PPARδ agonists results in transcriptional and posttranscriptional and the use of PPARα agonists in posttranscriptional induction of ICAM-1 expression in non-stimulated, quiescent human endothelial cells. Furthermore, the effects of PPAR agonists may depend on the activation status of endothelial cells, and this status may dictate pro-inflammatory versus anti-inflammatory responses. Therefore, these findings may influence the future development and application of PPAR agonists in the clinic.

## Abbreviations

HUVEC: Human Umbilical Vein Endothelial Cell
ICAM-1: Intercellular Adhesion Molecule-1
PPAR: Peroxisome Proliferator Activated Receptors
VCAM-1: Vascular Cell Adhesion Molecule-1
